# Bidirectional screening and treatment outcomes of diabetes mellitus (DM) and Tuberculosis (TB) patients in hospitals with measures to integrate care of DM and TB and those without integration measures in Malawi

**DOI:** 10.1186/s12879-021-07017-3

**Published:** 2022-01-04

**Authors:** John L. Z. Nyirenda, Dirk Wagner, Bagrey Ngwira, Berit Lange

**Affiliations:** 1 University Hospital Freiburg. Medical Faculty. University of Freiburg, Freiburg, Germany; 2 The Polytechnic College, University of Malawi, Blantyre, Malawi; 3 Helmholtz Centre for Infectious Research, Epidemiology, Braunschweig, Germany; 4 Public Health Department, Faculty of Applied Sciences, University of Livingstonia, Mzuzu, Malawi

**Keywords:** Tuberculosis, Diabetes mellitus, Integrated healthcare, Comorbidity, Bidirectional screening, Treatment loss to follow up

## Abstract

**Introduction:**

There are efforts in low and middle-income countries (LMICs) to integrate Tuberculosis (TB) and Diabetes mellitus (DM) healthcare services, as encouraged by WHO and other international health organizations. However, evidence on actual effect of different integration measures on bidirectional screening coverages and or treatment outcomes for both diseases in LMICs is scarce.

**Objectives and methods:**

Retrospective chart review analysis was conducted to determine effects of integrated care on bidirectional screening and treatment outcomes for both TB patients and people with DM (PWD) recruited in eight Malawian hospitals. Data of ≥ 15 years old patients registered between 2016 to August 2019 were collected and analysed.

**Results:**

557 PWDs (mean age 54) and 987 TB patients (mean age 41) were recruited. 64/557 (11.5%) PWDs and 105/987 (10.6%) of TB patients were from an integrating hospital. 36/64 (56.3%) PWDs were screened for TB in integrated healthcare as compared to 5/493 (1.0%) in non-integrated care; Risk Difference (RD) 55.2%, (95%CI 43.0, 67.4), P < 0.001, while 10/105 (9.5%) TB patients were screened for DM in integrated healthcare as compared to 43/882 (4.9%) in non-integrated care; RD 4.6%, (95%CI − 1.1, 10.4), P = 0.065. Of the PWDs screened, 5/41 (12.2%) were diagnosed with TB, while 5/53 (9.4%) TB patients were diagnosed with DM. On TB treatment outcomes, 71/508 (14.8%) were lost to follow up in non-integrated care and none in integrated care were lost to follow-up; RD − 14.0%, (95%CI: − 17.0,-11.0), p < 0.001. Among PWDs, 40/493 (8.1%) in non-integrated care and 2/64 (3.1%) were lost to follow up in integrated care; RD − 5.0%, (95%CI:-10.0, − 0.0); P = 0.046. After ≥ 2 years of follow up, 62.5% PWDs in integrated and 41.8% PWDs in non-integrated care were retained in care, RD 20.7, (95%CI: 8.1, 33.4), P = 0.001.

**Conclusion:**

We found higher bidirectional screening coverage and less loss to follow-up in one centre that made more efforts to implement integrated measures for TB and DM care than in 7 others that did not make these efforts. Decisions on local programs to integrate TB/DM care should be taken considering currently rather weak evidence and barriers faced in the local context as well as existing guidelines.

**Supplementary Information:**

The online version contains supplementary material available at 10.1186/s12879-021-07017-3.

## Introduction

### Background

Studies have shown that the risk of developing active tuberculosis (TB) is three times higher in persons with Diabetes mellitus (DM); RR 3.0, 95%CI (2.3, 4.3), than in persons without DM [1–4]. According to World Health Organisation (WHO) TB screening guidelines, uncontrolled DM doubles the risk of TB treatment failure, relapse and death [1], and both DM type 1 and DM type 2 (T2DM), are risks factors for the development of active TB with T2DM accounting for more than 90% of TB cases attributed to DM [5]. Although studies are still being conducted to better understand the immunological mechanisms of susceptibility of TB among people with Diabetes mellitus (PWD), several factors [6] in relation to complex natural history of TB progression [7] have already been endorsed that would increase the risk of TB among PWDs.

With wide variations between countries and regions that ranges from 1.9% to 45%, the average global DM prevalence is estimated at 16% [8, 9]. Further on TB and DM comorbidity, a meta-analysis that was conducted in 2018 found 5.13% (95% CI 4.34–5.92) pooled prevalence of TB among DM patients in Africa [10]. On the other hand, Peer et al. 2017 [11] reasoned that DM prevalence in the continent might be underestimated since the prevalence of undiagnosed DM is between 50.7 and 75.1% in LMICs.

The higher risk of developing TB in DM patients and the challenges of diabetic TB patients in the treatment and care of both diseases have been well recognized, hence integrated approach for TB/DM health care is recommended by WHO, The International Union against Tuberculosis and Lung Diseases (IUATLD) and The International Diabetes Federation (IDF) [12–14]. Integration is aimed at improving the service in relation to efficiency and quality, thereby maximising use of resources and opportunities through; promoting access to care for NCD patients, maximizing efficiency given the severe human resource shortages, and replicate strong infectious diseases outcomes for patients with other chronic conditions [15]. However, there is limited evidence on the effects of TB/DM integrated healthcare services especially in poor resource settings.

Due to increased burden of both NCDs and Infectious diseases, a few hospitals in Malawi started providing NCD care services between 2009 and 2017 with assistance from health development partners. However, NCDs were formally included in Health Sector Strategic Plan 2011–2016 in Malawi, and an NCDs Action Plan 2012–2016 was developed in the Ministry Of Health (MoH) [16] to facilitate adoption of the integrated approach to delivery of NCD services [16–18] in secondary level care hospitals in the country. This small landlocked country with approximately 19 million people [19–21] has 5.6% DM prevalence, 33% prevalence of hypertension among the adults aged between 25 to 64 years [22], and an estimated 41% of the identified PWDs were undiagnosed [23]. Furthermore, the country is ranked among top 20 global countries with the highest estimated numbers of incident TB cases among people living with HIV; 88 (55–129) per 100,000, and has an estimated TB incidence of 181 (113–265) per 100,000 in general population [24]. However, in Malawi, like other TB high incidence settings, evidence of the actual effect of integrated NCD/TB programs on patients is still missing. Therefore, this study was conducted in Malawi to investigate bidirectional screening and treatment outcomes of DM and TB patients in hospitals with measures to integrate care of DM and TB as compared with those without integration measures. In this study, integration of TB and DM health care was defined as a variety of managerial or operational changes to health systems to bring together inputs, delivery, management and organisation of TB and DM service functions [25] as well as different kinds of DM and TB services or operational programs or activities joined together to ensure and perhaps maximize collective outcomes for both DM and TB Patients. In Malawi, the integration efforts of DM and TB has been well described and categorized within the complex model where NCDs and HIV and TB services are incorporated in one clinic, offered by the same healthcare workers under one roof [15].

### Study objectives

The aim of the study was to determine the effects of integrated care on bidirectional screening and treatment outcomes for both TB patients and people with DM recruited from 2016 to 2019 in eight Malawian hospitals through retrospective chart review analysis. In this study, we investigated bidirectional screening coverage, proportion of DM cases among TB patients and proportion of TB cases among people with DM, described effect of integration on TB treatment success, diabetic complications, treatment loss to follow-up for both diseases, retention into DM care and mortality of TB patients and people with DM.

### Study variables

As stated on the objectives above, this study assessed the following variables: TB screening coverage among people with DM and vice versa, PWDs retention on care and treatment adherence, TB treatment outcomes as defined by WHO [26], and DM complications related to uncontrolled DM by comparing an integrating hospital with non-integrating hospitals.

## Methods

### Study design, setting and study population

We conducted a retrospective chart review analysis for patients’ data from an integrated chronic care clinic at Neno Hospital [15], and seven other hospitals that are operating TB and DM care services on the traditional approach. The study was conducted at secondary level healthcare in a poor country where 84% of the population lives in the rural areas as compared to 16% in urban centres [27]. This study was implemented within a quasi-experimental study and policy analysis research that is being conducted in six government district hospitals namely, Kasungu, Dowa, Bwaila (Lilongwe), Ntcheu, Neno and Mzuzu Health Center; and two Christian Health Association of Malawi (CHAM) hospitals namely, David Gordon Memorial (DGM) in Rumphi district and Embangweni Mission in Mzimba district. Retrospective data of patients aged ≥ 15 years old between January 2016 to August 2019 were collected by using hypertension and DM and TB treatment records.

### Sample size calculation and sampling method

**Fig. 1:**
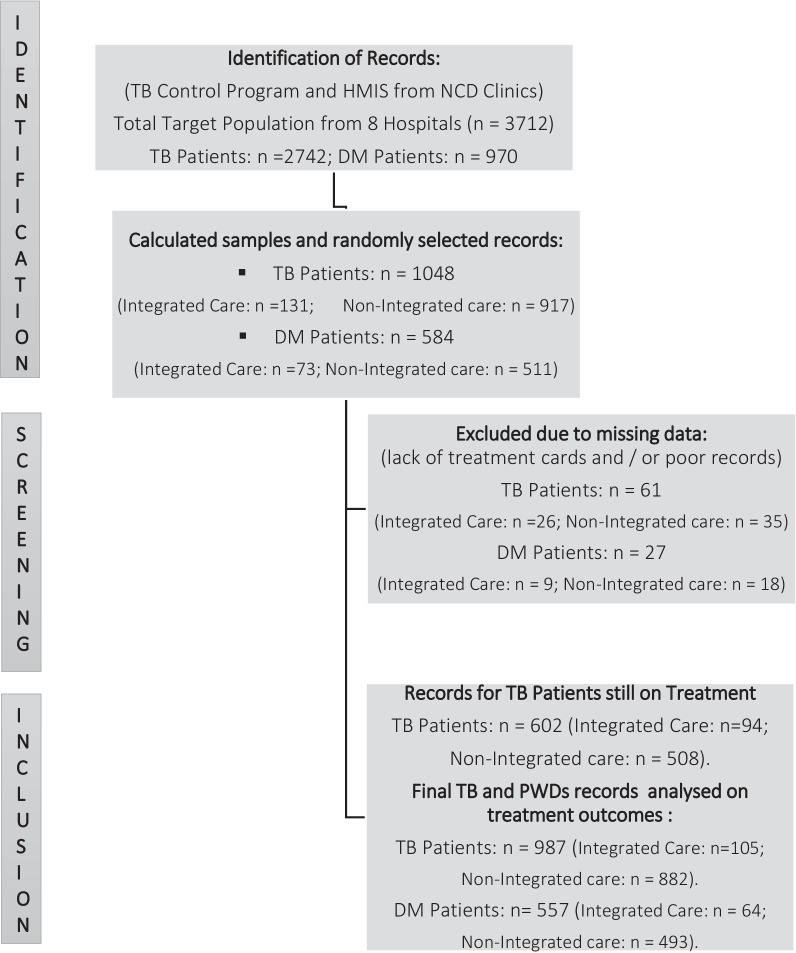
STROBE Flow Diagram showing the target population, participants' selection and attrition, screening, and finally analysed patients' records for the period between 2016 and 2019 in the study hospitals

Two sample proportions commands were applied in Stata Version 15.1 to calculate a sample size which was stratified by integrated and non-integrated care on a ratio of 1:7. We assumed 0.05 significant level, 0.90 power, 40% and 45% bidirectional screening coverages in non-integrated care setting for DM and TB respectively, and 60% for both TB and DM in integrated care. The overall sample size for DM patients was 584 and for TB patients was 1048. As shown on Fig. 1, patients were selected through simple random sampling method from the list of the available records. This method was deemed appropriate since the list of the participants were available at each study site. When data was missing for a randomly selected patient identification, a replacement was obtained from the remaining records following the same process. However, due to limited available records and missing data, more records of both TB patients and PWDs who were still on treatment were included.

### Data sources, data collection and management

Data were collected in August 2019—September 2019 by using a checklist that were developed from the data elements adopted on the DM and hypertension treatment cards and TB treatment cards that are used in hospitals in Malawi. However, data on diagnostics tests that were performed to confirming the cases was not collected since such information is not recorded on the cards, and high proportion of data from patients who were still on treatment were collected due to missing data or lack of data on the old records from respective sites.

After development of data collection tools, research assistants were selected and trained and coordinated by the principal investigator to facilitate data collection exercise. These trained research assistants worked collaboratively with laboratory managers, Health Management Information System Officers, TB officers and NCDs coordinators in respective study sites. Android tablets with the digital version of Open Data Kit (ODK) were used to collect and organise the survey data, allowing for immediate data validation in the field [28].

### Statistical analysis

Descriptive statistics in univariate analysis were performed and thereafter bivariate and multivariate analyses were performed to analyse the risk differences (RD) in variables between the two settings. Frequency tables, proportions and risk differences of the key findings were performed. After descriptive data analysis, age and sex were assessed for confounding by applying multinomial logistics regression (mlogit) with survey data setting in Stata 15.1 [29, 30]. Regressions were performed to assess the effects of sex and age on the association between the treatment outcomes and complication on their relationship with integrated care. Thereafter, calculated coefficients in the performed models were transformed to risk ratios and later converted into risk differences by direct commands in Stata 15.1.

### Ethical considerations and data protection

The study followed the ethical principles as outlined in the World Medical Association (WMA) declaration [31, 32]. The study was approved by both Ministry of Health in Malawi through National Health Sciences Research Committee (NHSRC) (approval number 2187), and by the ethical committee of the University of Freiburg (approval reference number 29/20). Data were stored in an encrypted online database, from where they were exported via excel file to Stata version 15.1 for statistical analysis. Data were anonymised before analysis.

## Results

### General patients’ characteristics and comorbidities

As shown on Fig. 1, records for 557 DM patients and 987 TB Patients were analysed in this study. 10.6% (n = 105) of the total 987 TB patients, and 11.5% (n = 64) of the total 557 PWDs were treated in the integrated clinic. There was a male predominance in the TB patient group (69.5%; n = 686) and a female predominance in the PWDs (58.0%; n = 323), which was similar in both the integrated and non-integrated setting.

TB patients were younger than PWDs (mean age 41 and 54 years, respectively) as shown on Table 1 and in Additional file 1: Table S1. 107/557 (19.2%) of the PWDs had Type 1 diabetes, 39/987 (4.0%) of the TB patients had MDRTB; see Table 2. 259/557 (46.5%) PWDs were hypertensive, 9.2% of PWDs had cardiovascular diseases whereas renal diseases were the least prevalent condition among PWDs (1.4%). On risk factors, 8/463 (1.7%) of the PWDs were smokers, 15/455 (3.3%) consumed alcohol, 89/198 (45.0%) consumed fruits and vegetables less than two times a week and 91/221 (41.2%) make less than two 30-min exercise sessions per week, respectively. Differences between integrated and non-integrated hospitals on PWDs performing daily 30 min of exercises involving planned activities such as jogging or walking sessions performed in the week as defined by WHO [33] or consumption of at least three or more times of fruits and vegetable portions per week were not significant, of course, with high percentages of missing records for these variables as seen on Table 2.

**Table 1:** Background characteristics of patients seen at both integrated and non-integrated hospitals

A. Characteristics of Tuberculosis Patients				
Total TB Patients and stratified by integration status		Totals: n = 987 (100%)	Integrated care: n = 105 (10.6%)	Non-Integrated care: n = 882 (89.4%)
TB Patients' Categories	New	863 (87.4%)	90 (85.7%)	773 (87.6%)
	Failed	4 (0.4%)	0 (0.0%)	4 (0.5%)
	Treatment after loss to follow up	10 (1.0%)	1 (1.0%)	9 (1.0%)
	Relapse	96 (9.7%)	14 (13.3%)	82 (9.3%)
TB Type	Extra-Pulmonary	263 (26.6%)	50 (47.6)	213 (24.1%)
	Pulmonary	724 (73.4%)	55 (52.4%)	669 (75.9%)
MDRTB	Yes	39 (4.0%)	1 (1.0%)	38 (4.3%)
	No	948 (96.0%)	104 (99.0%)	844 (95.7%)
Sex	Female	301 (30.5%)	39 (37.1%)	262 (29.7%)
	Male	686 (69.5%)	66 (62.9%)	620 (70.3%)
**TB Patients with available information on age**		** (N = 932)**	** (n = 97)**	** (n = 835)**
Age distribution	Mean, Median (Min; 1stq; 3rdq; max)	41.1,38.0 (15,30,48,89)	47.1,43.0 (15,38,60,83)	40.4, 38.0 (15,29,46,89)
	≤ 25 years	150 (16.1%)	12 (12.4%)	138 (16.5%)
	26–40 Years	398 (42.7%)	31 (32.0%)	367 (44.0%)
	41–49 Years	169 (18.1%)	18 (18.6%)	151 (18.1%)
	50–64 Years	136 (14.6%)	17 (17.5%)	119 (14.3%)
	≥ 65 years	79 (8.5%)	19 (19.6%)	60 (7.2%)

B. Characteristics of Diabetes mellitus Patients				
Total PWDs and stratified by integration status		Totals: n = 557 (100%)	Integrated care: n = 64 (11.5%)	Non-Integrated care: n = 493 (88.5%)
DM Type	Type 2	450 (80.8%)	50 (78.1%)	400 (81.1%)
	Type 1	107 (19.2%)	14 (21.9%)	93 (18.9%)
Years in Care Since Enrolment	≤ 1 Year	311 (55.8%)	24 (37.5%)	287 (58.2%)
	2 to 3 Years	128 (23.0%)	28 (43.8%)	100 (20.3%)
	4 to 5 Years	52 (9.3%)	5 (7.8%)	47 (9.5%)
	6 to 7 Years	40 (7.2%)	3 (4.7%)	37 (7.5%)
	≥ 8 Years	26 (4.7)	4 (6.3%)	22 (4.5%)
Sex	Female	323 (58.0%)	35 (54.7%)	288 (58.4%)
	Male	234 (42.0%)	29 (45.3%)	205 (41.6%)
**PWDs with available information on age**		** (N = 496)**	** (n = 64)**	** (n = 432)**
Age distribution	Mean, Median	53.6,55	52.4, 51.5	53.8, 55
	(Min; 1stq; 3rdq; max)	(15,43,65,87)	(16,41.5,63,87)	(15,44,65,87)
	≤ 25 years	26 (5.2%)	4 (6.3%)	22 (5.1%)
	26–40 Years	77 (15.5%)	11 (17.2%)	66 (15.3%)
	41–49 Years	82 (16.5%)	12 (18.8%)	70 (16.2%)
	50–64 Years	186 (37.5%)	25 (39.1%)	161 (37.3%)
	≥ 65 years	125 (25.2%)	12 (18.8%)	113 (26.2%)

Background characteristics of both TB patients and people with DM who were seen at the study hospitals between 2016 and 2019

**Table 2:** Comorbidities among TB patients and People living with DM and DM related complications

HIV Infections and DM conditions among TB Patients				
Variables	Category	Totals	Integrated	Non-Integrated
		N = 987 (100%)	n = 105 (10.6%)	n = 882 (89.4%)
*HIV Infected	Yes	372/908 (41.0%)	50/104 (48.1%)	322/804 (40.1%)
	No	536/908 (59.0%)	54/104 (51.9%)	482/804 (59.9%)

HIV and Hypertension Comorbidity and Risk factors related to Complications among PWDs				
Variables	Category	Totals	Integrated	Non-Integrated
		N = 557 (100%)	n = 64 (11.5%)	n = 493 (88.5%)
HIV Infected	Yes	34 (6.1%)	10 (15.6%)	24 (4.9%)
	No	523 (93.9%)	54 (84.4%)	469 (95.1%)
Hypertension (HTN)	Yes	259 (46.5%)	27 (42.2%)	232 (47.1%)
	No	298 (53.5%)	37 (57.8%)	261 (52.9%)
Cardiovascular Diseases	Yes	51 (9.2%)	9 (14.1%)	42 (8.5%)
	No	506 (90.8%)	55 (85.9%)	451 (91.5%)
Peripheral Heart Diseases	Yes	13 (2.3%)	5 (7.8%)	8 (1.6%)
	No	544 (97.7%)	59 (92.2%)	485 (98.4%)
Retinopathy	Yes	14 (2.5%)	9 (14.1%)	5 (1.0%)
	No	543 (97.5%)	55 (85.9%)	488 (99.0%)
Renal Disease	Yes	8 (1.4%)	7 (10.9%)	1 (0.2%)
	No	549 (98.6%)	57 (89.1%)	492 (99.8%)
Stroke /TIA	Yes	15 (2.7%)	10 (15.6%)	5 (1.0%)
	No	542 (97.3%)	54 (84.4%)	488 (99.0%)
Neuropathy	Yes	32 (5.8%)	16 (25.0%)	16 (3.3%)
	No	525 (94.2%)	48 (75.0%)	477 (96.7%)
Sexual dysfunction	Yes	9 (1.6%)	7 (10.9%)	2 (0.4%)
	No	548 (98,4%)	57 (89.1%)	491 (99.6%)
*Smoking ( PWDs with available information = 463)	Yes	8 (1.7%)	2 (3.2%)	6 (1.5%)
	No	455 (98.3%)	61 (96.8%)	394 (98.5%)
*Alcohol Consumption (PWDs with available information = 455)	Yes	15 (3.3%)	3 (4.9%)	12 (3.1%)
	No	440 (96.7%)	58 (95.1%)	382 (97.0%)
*Fruit / Vegetable Portions per week (PWDs with available information = 198)	≥ Three Times	109 (55.0%)	5 (25.0%)	104 (58.4%)
	≤ Two Times	89 (45.0%)	15 (75.0%)	74 (41.6%)
*Frequency of daily 30 min of exercise per week (PWDs with available information = 221)	≥ Three Times	130 (58.8%)	19 (73.1%)	111 (56.9%)
	≤ Two Times	91 (41.2%)	7 (26.9%)	84 (43.1%)

*Total assessed records were less that total sampled due to missing data. The missing records were not included in final analysis

Comorbidities and DM related complications for the patients reported at the study hospitals between 2016 -2019 in Malawi.

**Table 3:** Screening coverages and TB/ DM Comorbidities among TB patients and People with DM

TB and DM Bidirectional Screening						
A. Tuberculosis Patients						
Variables	Category	Totals n = 987 (100%)	Integrated site n = 105 (10.6%)	Non-Integrated sites n = 882 (89.4%)	RD (%) (95%CI)	
DM Screening and DM Comorbidities among TB Patients						
TB patients screened for DM		53 (5.4%)	10 (9.5%)	43 (4.9%)	4.6% (-1.1,10.4)	
Diagnosed DM Cases amongst the screened TB Patients, and of the total TB patients						
DM among screened TB Patient		5/53 (9.4%)	1/10 (10.0%)	4/43 (9.3%)		
DM among total TB Patients		5/987 (0.5%)	1/105 (1.0%)	4/882 (0.5%)		

B. TB Screening and TB Comorbidities among People with Diabetes mellitus (PWD)						
Variables	Category	Totals N = 557 (100%)	Integrated n = 64 (11.5%)	Non-Integrated n = 493 (88.5%)	Risk Difference (%) (95%CI)	
Screening of TB among People with Diabetes mellitus (PWD)						
PWD screened for TB		41 (6.6%)	36 (56.3%)	5 (1.0%)	55.2 (43.0,67.4)	
Diagnosed TB Cases amongst the screened people with DM, and of the total People with DM						
Pulmonary		3 /41 (7.3%)	1/36 (2.8%)	2/5 (40.0%)		
Extra-Pulmonary		2/41 (4.9%)	0	2/5 (40.0%)		
TB cases of Screened PWD		5/41 (12.2%)	1/36 (2.8%)	4/5 (80.0%)		
TB cases of total recruited PWD		5/557 (0.9%)	1/64 (1.6%)	4/493 (0.8%)		

DM; Diabetes mellitus, PWD; People with DM, TB; Tuberculosis

Screening coverages and comorbidities among TB Patients and People with Diabetes recorded between 2016 -2019. Abbreviations: RD = Risk difference; PWD = People with Diabetes mellitus

### TB and DM Screening coverage of both PWD and TB cases

On Table 3, 10/105 (9.5%) TB patients were screened for DM in integrated care while 43/882 (4.9%) were screened for DM in non-integrated care setting, RD 4.6, 95%CI (− 1.1, 10.4), P = 0.065 respectively. Of the total TB patients screened, 5/53 (9.4%) were diagnosed with DM. Among PWDs, 36/64 (56.3%) were screened for TB in integrated care setting as compared to 5/493 (1.0%) in the traditional non-integrated care delivery system care RD 55.2%, (95%CI 43.0, 67.4), P < 0.001, and 5/41 (12.2%) of the screened DM patients were diagnosed with TB.

### Treatment outcomes, glucose monitoring and retention into care

As shown on Table 4, 71/508 (14.0%) TB patients were lost to follow-up in non-integrated care hospitals, while in an integrated care, none were lost to follow-up in integrated care; RD -14.0%, (95%CI: -17.0,-11.0), p < 0.001, and TB treatment success was 92.5% in integrated cares, while in non-integrated care the treatment success was 78.5%, RD 14, (95%CI 7.6, 20.4); P = 0.001.

Among people with DM, 2/64 (3.1%) experienced loss to follow up in integrated care and 40/493 (8.1%) in non-integrated care, RD -5.0%, (95%CI: -10.0, -0.0%); P = 0.046.

On retention into care by PWD, 52/64 (81.3%) PWD in integrated care were in care for ≤ 3 years while in traditional non- integrating setting 387/493 (78.5%), RD 2.8%, 95%CI (-7.5, 13.0), P = 0.630 were recorded. Furthermore, aggregated data on Table 4 shows that after ≥ 2 years of follow up, 62.5% PWDs in integrated and 41.8% PWDs in non-integrated care were retained in care, RD 20.7, (95%CI: 8.1, 33.4), P = 0.001.

**Table 4:** Treatment Loss to follow up and treatment outcomes for TB patients, and Treatment Loss to follow up, Glucose monitoring and Retentions into Care by people living with DM

Treatment outcomes and DM Patients retention:					
A. Tuberculosis Patients					
Variables	Totals n = 602 (100%)	Integrated n = 94 (15.6%)	Non-Integrated n = 508 (84.4%)	RD (%) (95%CI)	RD (%) (95%CI) as controlled by both Sex and Age
Treatment Outcomes					
Treatment Loss to follow up	71 (11.8%)	0	71 (14.0%)	-14.0 (-17.0,-11.0)	-
Treatment Success	486 (80.7%)	87 (92.5%)	399 (78.5%)	14.0 (7.6,20.4)	14.3 (10.7,17.8)
Died	45 (7.5%)	7 (7.4%)	38 (7.5%)	-0.1 (-3.2,3.1)	-0.4 (-3.5,2.8)
B. People with Diabetes mellitus					
	Total: N = 557 (100%)	Integrated care: n = 64 (11.5%)	Non-Integrated care: n = 493 (88.5%)	RD (%) (95%CI)	RD (%) (95%CI) as controlled by both Sex and Age
Treatment outcomes Outcome					
On treatment & transferred out	508 (91.2%)	61 (95.3%)	447 (90.7%)	4.6 (-1.1,10.4)	5.0 (-1.1,11.1)
Loss to follow up & stopped	42 (7.5%)	2 (3.1%)	40 (8.1%)	-5.0 (-10.0,-0.0)	-5.3 (-10.4,0.0)
Died	7 (1.3%)	1 (1.6%)	6 (1.2%)	0.4 (-2.8, 3.5)	0.3 (-3.1, 3.7)
Quarterly FBG Checks (n, %)					
FBS checked at 6 months	153 (27.5%)	27 (42.2%)	126 (25.6%)	16.6 (4.7,28.5)	16.4 (4.5,28.4)
FBS checked at 12 months	138 (24.8%)	32 (50.0%)	106 (21.5%)	28.5 (15.3,41.7)	28.1 (15.4,40.9)
Years in Care Since Enrolment (n, %)					
≤ 1 Year	311 (55.8%)	24 (37.5%)	287 (58.2%)	-20.7 (-33.4,-8,1)	
2 to 3 Years	128 (23.0%)	28 (43.8%)	100 (20.3%)	23.5 (10.8,36.1)	
4 to 5 Years	52 (9.3%)	5 (7.8%)	47 (9.5%)	-1.7 (-8.8,5.4)	
6 to 7 Years	40 (7.2%)	3 (4.7%)	37 (7.5%)	-2.8 (-8.5,2.9)	
≥ 8 Years	26 (4.7)	4 (6.3%)	22 (4.5%)	1.8 (-4.4,8.0)	

Showing treatment outcomes for TB patients and treatment loss to follow, glucose monitoring and retentions into care among people with DM

On blood glucose monitoring, 27/64 (42.2%) PWDs were checked for fasting blood glucose (FBG) assessment at 6 months at an integrating hospital, and 126/493 (25.6%) were checked at non-integrating hospitals RD 16.6%, (95%CI: 4.7,28.5), P = 0.005, while at 12 months; 32/64 (50.0%) were checked at integrating hospital while 106/493 (21.5%) were checked at non-integrating hospitals, 95%CI (15.3,41.7), P < 0.001, respectively (table 4). Additionally, 423/493 (85.8%) of PWD had their weights checked after 6 months into care at an integrated care hospital, while 28/64 (43.8%) were checked during the same period in non-integrated care hospitals.

## Effects of integrated care on Treatment Success and mortality among TB patients, and Treatment Loss to follow up and or Stopped and quarterly FBG checks among people with DM as stratified as controlled by sex and age

After controlling for age and sex, as shown on Table 5, we observed that both sex and age of the patients did not significantly affect the association that was observed between integration status and bidirectional screening and treatment outcomes. However, on screening, the calculated crude risk ratio (RR) was RR1.9, 95%CI 0.2,20.1), while after controlling for sex and age, RR was increasing, but not significantly different as shown by RR2.2, (95%CI: 0.2,24.5). On the other hand, among PWDs, we found that PWDs were more likely to have FBG checked at 12 months in integrated care than in hospitals without integrating measures; RR2.3, 95%CI 1.2,4.5, and the results were statistically significant. Like results in screening, the crude RR did not significantly change after controlling for sex and age.

**Table 5:** Showing association between integrated care and Treatment Success and mortality among TB patients, treatment loss to follow up and or stopped and Quarterly FBG checks among DM patients as controlled by sex, age and both age and sex

Effects of Integrated Care on Treatment Loss to follow up and or Stopped and quarterly FBG checks among DM patients				
Variable	Integrated Healthcare RR (95%CI)	RR (95%CI) controlled by Sex	RR (95%CI) as controlled by age	RR (95%CI) as controlled by both Sex and Age
Treatment Outcomes among TB patients				
Treatment Success	1.8 (1.2,2.7)	1.8 (1.2,2.7)	1.8 (1.2,3.0)	1.8 (1.2,2.8)
Died TB Patients	1.6 (0.1,24.6)	1.5 (0.1,29.1)	1.6 (0.1,26.2)	1.6 (0.1,30.1)
Probability of screening TB patients for DM				
Screening Likelihood	1.9 (0.2,20.1)	1.9 (0.2,19.4)	2.1 (0.2,25.0)	2.2 (0.2,24.5)
Treatment Outcomes among DM patients				
Loss to follow up, stopped	0.4 (0.1,1.7)	0.4 (0.1,1.7)	0.4 (0.1,1.6)	0.4 (0.1,1.6)
Quarterly Fasting Blood Glucose (FBG) Checks				
FBS checked at 6 months	1.6 (1.0,2.7)	1.7 (1.1,2.8)	1.6 (1.0, 2.7)	1.7 (1.0, 2.7)
FBS checked at 12 months	2.3 (1.2,4.5)	2.4 (1.3,4.6)	2.3 (1.2,4.3)	2.4 (1.3,4.3)

## Discussion

With an increasing global prevalence of TB and DM comorbidity, integrated approaches to care for effective management of both diseases especially in LMICs where heath systems are weak, TB is endemic, and where 80% of global type 2 DM are found [7] have been recommend. In this study, we found that 9.4% of the screened TB patients were living with DM which is suggesting high prevalence of TB among DM patients and high DM among TB patients than in general population in Malawi where prevalence of DM is approximately 5.6%, hypertension is 33%, HIV infections is 9.0% [18, 34], and that of TB is 181 (113–265) per 100,000 [24]. Furthermore, our findings support results from several other observational studies that were conducted in a few LMICs countries such as South Africa, Romania, China, Peru, Indonesia and India [12, 13, 35–40] where 10.9%, 12.3, 14.0%, 19.7%, 14.0% and 24% prevalence of DM among TB patients were reported respectively. These results support the estimated high global TB and DM comorbidities where one million out of 9.6 million annual TB cases (10.4%) are estimated to be living with DM [6]. With such high prevalence of TB and DM comorbidity, WHO, IUATLD and IDF [12–14] are recommending adoption of TB/DM integrated approach to services delivery, however, each country should consider specific epidemiology of both TB and DM and strength of health systems.

Screening coverage for DM among TB patients in integrated settings was nearly 2 times that in non-integrated settings, but this only meant an overall increase from 4.9 to 9.5% and is less than what would be needed to generate wider impact. Nevertheless, low screening coverage and low yields, in our study, might have been attributed to barriers in implementation of integrated care program. For instance, two implementation research studies from Mexico and China [39, 40] found that shortages of human and materials resources are among factors that affected the outcomes of the integrated care services in their pilot sites. These challenges are inevitable in Malawi, a poor country, like other countries in Sub-Saharan Africa (SSA), with a critical shortage of healthcare workers [41]. In addition to human resource shortages, observational studies that were conducted in India [42, 43] found that other factors such as patients perspectives, recording system and lack of supplies and laboratory equipment like glucometers and testing strips may affect implementation of integrated screening services in hospitals. Thus, governments in Africa are advised to consider improving health systems for effective management of the increasing numbers of NCDs such as DM [44]. Irrespective of the underlying reasons for low screening coverage of DM in TB patients, our results indicate that within implementation of integrating care it is important to measure screening coverage and make efforts to increase it.

On treatment outcomes, we found less treatment loss to follow up in integrated care than in non-integration, and better TB treatment success in integrated care than in traditional non-integrated care even after controlling for both sex and age. The findings agree with those of an implementation research study that was conducted in Mexico [40] which showed that joint management of TB and DM comorbid patients reported lower proportions of treatment loss to follow up and increased treatment success than in non-integrated care system. In another study that was conducted in Taiwan [45], it was found that enhanced case management of PWDs on a Pay-4-Performacne program (p4p) contributed to reduced annual incidence rate of TB to 137.5/100,000 in DM-p4p group as compared to 259.9/100,000 in DM-non-p4p. Furthermore, the same study reported an improvement in TB treatment outcomes among TB patients with DM in DM-p4p program as compared to DM-non-p4p program.

In this study, retrospective follow up of PWDs for 12 months into care program showed high retention rate among PWDs in integrated care than in non-integrated care. Studies have shown that retention into care helps to ensure glucose monitoring and early identification of complications. For instance, Harries et al. [3] found that inadequate glucose monitoring, under diagnosis, inadequately treated and poorly controlled DM appears to be a much greater threat to TB care and prevention than previously realized, and the authors further reported that the problem of uncontrolled glucose among DM patients is critical in poor countries.

On DM related complication, we found more records of complications in the integrated hospital than in non-integrating hospitals a situation which could suggest reverse causality [46] since integrated care might have contributed to increased and improved diagnosis of DM complications in the integrated clinic. Therefore, the observed association might have been attributed to effects of integrated clinic services where more conditions could be identified than in non-integrated hospitals. On the other hand, background differences in the study sites and study participants might have confounded the results.

During the study, one hospital was integrating, and this hospital was compared against seven other hospitals which applies traditional or non-integrated method. This integrating hospital is supported by an international organisation; Partners in Health (PIH), which has contributed to health systems strengthening through capacity building by providing materials and employing of additional healthcare workers at the Integrated NCD clinic [15]. Therefore, if TB and DM integrated care services are to be scaled up to other hospitals in Malawi, lessons from this piloting hospital should be carefully considered. Furthermore, considerations should be made on the actual activities to integrate, availability of supplies and staff at a standard district hospital, level of integration within the health system and how the impact of the integration on the efficiency of services delivery shall be monitored [47].

## Study strengths and limitations

The introduction of the treatment cards made this study possible. However, hospitals experienced shortage of treatment cards such that patients, in some instances, were being recorded in improvised notebooks, and that these cards did not have all the data elements on TB patient records. For instance, data on diagnostics tests that were performed to confirming the cases was not collected since such information is not recorded on the cards. Furthermore, high proportion of data from patients who were still on treatment were collected due to missing data of the old records, and that the improvised notebooks could not capture all needed data elements as indicated on the card. In addition to shortage of treatment cards, we found many cards with blank spaces which contributed to high proportion of missed data. Apart from other general standards for secondary level of care at district hospitals in Malawi, Neno had additional staff employed by the NGO, which is supporting the integrated NCDs clinic. Furthermore, small sample size especially from the integrated care since one hospital was integrating at the time when data collection was performed, and that socio-economic characteristics of both TB patients and PWDs in all non-integrating hospitals might not have been like those at the integrating hospital. Additionally, we included more TB patients who were still on treatment due to availability of records and missing data in the past records, such that less TB records were analysed on treatment outcomes.

## Conclusions

We found that in one setting with efforts of integrated care compared to seven without these efforts TB screening for DM patients is increased, that treatment loss to follow up in both diseases is reduced, and treatment success among TB patients is improved with TB/DM integration measures, but only limited evidence on the increase of DM screening for TB patients. Therefore, decisions on hospitals to integrate TB/DM should be taken considering burden of the disease, and the currently rather weak evidence of an effect on bidirectional screening coverage and treatment outcomes of both diseases, and the barriers faced in the local context as well as existing guidelines.

## Supplementary Information

**Additional file 1: Table 1.** Supplementary Data on distribution of both TB patients and people living with DM as stratified by sex and age per study site.

## Data Availability

The datasets used and/or analysed during the current study are available from the corresponding author on reasonable request. We confirm that data generated or analysed during this study are included in tables and in a figure published in this article.
