# Supplementary material for: Bidirectional screening and treatment outcomes of diabetes mellitus (DM) and Tuberculosis (TB) patients in hospitals with measures to integrate care of DM and TB and those without integration measures in Malawi
Source: BMC Infect Dis. 2022 Jan 4;22:28. doi: 10.1186/s12879-021-07017-3 (PMC8725264; doi:10.1186/s12879-021-07017-3)
Supplement: Supplementary file 1 — Additional file 1: Table 1. Supplementary Data on distribution of both TB patients and people living with DM as stratified by sex and age per study site. [file 12879_2021_7017_MOESM1_ESM.docx]

| **Bidirectional screening and treatment outcomes of Diabetes mellitus (DM) and Tuberculosis (TB) patients in hospitals with measures to integrate care of DM and TB and those without integration measures in Malawi** | | | | | | | | | |
| --- | --- | --- | --- | --- | --- | --- | --- | --- | --- |
|  |  |  |  |  |  |  |  |  |  |
| Table 1: Supplementary Data on distribution of both TB patients and people living with DM as stratified by sex and age per study site | | | | | | | | | |
|  |  |  |  |  |  |  |  |  |  |
|  |  |  |  |  |  |  |  |  |  |
| **A. Distribution of TB patients as categorized by Sex and Age group characteristics for each study site** | | | | | | | | | |
|  |  |  |  |  |  |  |  |  |  |
| Site | Bwaila | DGM | Dowa | Embangweni | Kasungu | Mzuzu | Neno | Ntcheu | Total |
| **Sex** | **210** | **38** | **162** | **55** | **157** | **182** | **105** | **78** | **987** |
| Female | 64 | 14 | 52 | 29 | 32 | 41 | 39 | 30 | 301 |
| (%) | 30,5 | 36,8 | 32,1 | 52,7 | 20,4 | 22,5 | 37,1 | 38,5 | 30,5 |
| Male | 146 | 24 | 110 | 26 | 125 | 141 | 66 | 48 | 686 |
| (%) | 69,5 | 63,2 | 67,9 | 47,3 | 79,6 | 77,5 | 62,9 | 61,5 | 69,5 |
| *Pearson chi2(7) = 31.2950 P-value <0.001* | | | | | | | | | |
|  |  |  |  |  |  |  |  |  |  |
| **Age Groups** | **183** | **38** | **160** | **52** | **153** | **174** | **97** | **75** | **932** |
| ≤ 25 years | 37 | 2 | 28 | 9 | 18 | 31 | 12 | 13 | 150 |
| (%) | 20,2 | 5,3 | 17,5 | 17,3 | 11,8 | 17,8 | 12,4 | 17,3 | 16,1 |
| 26 -40 Years | 77 | 13 | 57 | 17 | 75 | 96 | 31 | 32 | 398 |
| (%) | 42,1 | 34,2 | 35,6 | 32,7 | 49,0 | 55,2 | 32,0 | 42,7 | 42,7 |
| 41- 49 Years | 31 | 11 | 32 | 14 | 25 | 27 | 18 | 11 | 169 |
| (%) | 16,9 | 29,0 | 20,0 | 26,9 | 16,3 | 15,5 | 18,6 | 14,7 | 18,1 |
| 50 -64 Years | 27 | 6 | 24 | 8 | 28 | 16 | 17 | 10 | 136 |
| (%) | 14,8 | 15,8 | 15,0 | 15,4 | 18,3 | 9,2 | 17,5 | 13,3 | 14,6 |
| ≥ 65 years | 11 | 6 | 19 | 4 | 7 | 4 | 19 | 9 | 79 |
| (%) | 6,0 | 15,8 | 11,9 | 7,7 | 4,6 | 2,3 | 19,6 | 12,0 | 8,5 |
| *Pearson chi2(28) = 66.0378 P-value = 0.001* | | | | | | | | | |
|  |  |  |  |  |  |  |  |  |  |
|  |  |  |  |  |  |  |  |  |  |
| **B. Distribution of People living with Diabetes mellitus as categorized by Sex and Age group characteristics for each study site** | | | | | | | | | |
| Site | Bwaila | DGM | Dowa | Embangweni | Kasungu | Mzuzu | Neno | Ntcheu | Total |
| **Sex** | **126** | **17** | **108** | **79** | **72** | **33** | **64** | **58** | **557** |
| female | 76 | 10 | 60 | 47 | 40 | 17 | 35 | 38 | 323 |
| (%) | 60,3 | 58,8 | 55,6 | 59,5 | 55,6 | 51,5 | 54,7 | 65,5 | 58,0 |
| male | 50 | 7 | 48 | 32 | 32 | 16 | 29 | 20 | 234 |
| (%) | 39,7 | 41,2 | 44,4 | 40,5 | 44,4 | 48,5 | 45,3 | 34,5 | 42,0 |
| *Pearson chi2(7) = 2.9996 P-value= 0.885* | | | | | | | | | |
|  |  |  |  |  |  |  |  |  |  |
| **Age Groups** | **86** | **14** | **107** | **70** | **69** | **32** | **64** | **54** | **496** |
| ≤ 25 years | 4 | 0 | 7 | 4 | 1 | 0 | 4 | 6 | 26 |
| (%) | 4,7 | 0,0 | 6,5 | 5,7 | 1,5 | 0,0 | 6,3 | 11,1 | 5,2 |
| 26 -40 Years | 12 | 0 | 18 | 4 | 14 | 3 | 11 | 15 | 77 |
| (%) | 14,0 | 0,0 | 16,8 | 5,7 | 20,3 | 9,4 | 17,2 | 27,8 | 15,5 |
| 41- 49 Years | 15 | 0 | 16 | 10 | 9 | 7 | 12 | 13 | 82 |
| (%) | 17,4 | 0,0 | 15,0 | 14,3 | 13,0 | 21,9 | 18,8 | 24,1 | 16,5 |
| 50 -64 Years | 29 | 5 | 35 | 30 | 32 | 15 | 25 | 15 | 186 |
| (%) | 33,7 | 35,7 | 32,7 | 42,9 | 46,4 | 46,9 | 39,1 | 27,8 | 37,5 |
| ≥ 65 years | 26 | 9 | 31 | 22 | 13 | 7 | 12 | 5 | 125 |
| (%) | 30,2 | 64,3 | 29,0 | 31,4 | 18,8 | 21,9 | 18,8 | 9,3 | 25,2 |
| *Pearson chi2(28) = 52.0561 P-value = 0.004* | | | | | | | | | |
